# Illicit Tobacco in Lithuania: A Cross-Sectional Survey

**DOI:** 10.3390/ijerph17197291

**Published:** 2020-10-06

**Authors:** Vaida Liutkutė-Gumarov, Lukas Galkus, Janina Petkevičienė, Mindaugas Štelemėkas, Laura Miščikienė, Aušra Mickevičienė, Justina Vaitkevičiūtė

**Affiliations:** 1 Health Research Institute, Faculty of Public Health, Lithuanian University of Health Sciences, 44307 Kaunas, Lithuania; vaida.liutkute@gmail.com (V.L.-G.); lukas.galkus@lsmuni.lt (L.G.); janina.petkeviciene@lsmuni.lt (J.P.); mindaugas.stelemekas@lsmuni.lt (M.Š.); laura.miscikiene@lsmuni.lt (L.M.); 2 Department of Preventive Medicine, Faculty of Public Health, Lithuanian University of Health Sciences, 44307 Kaunas, Lithuania; ausra.mickeviciene@lsmuni.lt; 3 Department of Health Management, Faculty of Public Health, Lithuanian University of Health Sciences, 44307 Kaunas, Lithuania

**Keywords:** tobacco, cigarettes, illicit trade, smoking, Lithuania

## Abstract

Taxation policies are the most cost-effective measure to reduce overall tobacco consumption. However, cigarettes in Lithuania are among the cheapest in the European Union. The threat of the illicit trade is often used to compromise evidence-based policies, pricing policies particularly. The aim of this study was to determine the extent of illicit cigarette consumption in Lithuania and identify the main characteristics of illicit cigarette smokers. The national cross-sectional survey with direct observation of the latest purchased pack of cigarettes was conducted between August and September 2019. In total, 1050 smokers aged ≥18 were interviewed face-to-face. The illicit share of the total consumption of cigarettes per year was 10.7% with 9.7% of smokers showing or describing illicit cigarette packs compared to 17% reported by industry-funded studies. Older smokers, smokers with lower education and heavy smokers were more likely to regularly purchase illicit cigarettes. The average price of an illicit pack was almost two times lower than licit. Although the illicit trade of tobacco products is a serious policy challenge, the threat of an increase in illicit trade should not delay tobacco taxation improvements.

## 1. Introduction

World Health Organization (WHO) defines illicit trade in tobacco products as any practice or conduct relating to the production, shipment, receipt, possession, distribution, sale, or purchase that is prohibited by law and includes any practice or conduct intended to facilitate such activity [1]. It is estimated that the global illicit cigarette market accounts for about 11.6%, resulting in approximately $40.5 billion USD tax revenue lost per year [2].

Illicit cigarette trade poses a serious threat to public health because it undermines tobacco control policies and government’s authority; causes substantial loss in tax revenues; increases availability of tobacco products and results in cheaper prices, which can increase consumption; contributes to the funding of international criminal activities; allows tobacco industry (TI) to engage with policy makers and helps them to get media attention; and last, but not least, helps them to be perceived as a legitimate stakeholder in a policy debate [3,4,5,6,7,8,9,10].

Lack and even non-existence of public transparent data on illicit tobacco is a global challenge for tobacco control research [11,12]. The nature of the illicit trade makes it hard to monitor and track; therefore, reliable data is often very scarce. Such circumstances give the opportunity to the TI to fill existing gaps with their conducted or commissioned research on the topic. Though such research is long being questioned in Europe, the United States, Asia, Australia, Brazil, and South Africa [11,13,14], TI is still a major funding source of the data on illicit trade [10]. Philip Morris International (PMI) alone has pledged $48 million USD to fund research on illicit tobacco trade and granted 61 projects worldwide in 2016–2017. This includes two projects in Lithuania [15,16]. Given that many countries lack independent data on the illicit tobacco trade, an urgent need for counterbalance is evident.

In Lithuania, there is no regular monitoring of the extent of illicit trade, apart from the data on the seizure of cigarettes provided by enforcement authorities. Local policy makers, following the example of the European Commission, frequently use data compiled by the Klynveld Peat Marwick Goerdeler International Cooperative (KPMG), which has a longstanding relationship with the TI [17]. KMPG reports have always listed Lithuania among countries with the highest prevalence of illicit cigarettes. According to the latest (2018) report, Lithuania has one of the highest illicit cigarette consumption rates (17%) in the European Union (EU) [18]. Meanwhile, The Pricing Policies and Control of Tobacco in Europe Project (PPACTE) found that KPMG overestimated illicit trade in 11 out of 16 analysed countries [19].

It is known that illicit trade is more frequent in countries with a land or sea border with Non-European Economic Area countries [20]. Besides, Ukraine, Russia, Moldova, and Belarus are considered to be major suppliers of cheap and illicit cigarettes in Europe [19]. Lithuanian border with Belarus and Russia accounts for 60% of state borders. Due to the favourable geographical and economic (price differences in the region) conditions, Lithuania is a country of transit, transhipment, temporary storage, and preparation for further shipments of the contraband cigarettes to Western Europe. Together with Latvia, Croatia, Greece, and Bulgaria, Lithuania is listed among countries where illicit cigarettes have the highest penetration [21]. This adds to the evident urgent need of methodologically transparent independent estimates of illicit tobacco in Lithuania.

The aim of this study was to determine the extent of illicit cigarette consumption in Lithuania using direct methods and identify the main characteristics of illicit cigarettes smokers.

## 2. Materials and Methods

### 2.1. Study Sample and Data Collection

The survey was performed by the market research company Rotelas S.A. Face-to-face interviews including an on-site cigarette pack observation were conducted in the households between August and September 2019. A multistage, stratified, proportional sampling method was used. The sample was designed to represent the smoking population aged 18 and over in Lithuania. The sample size of 1000 and the ratio of men and women (3:1) in the sample were based on the research data of smoking prevalence in Lithuania [22]. The survey covered the whole country and was performed in all ten administrative regions of Lithuania. The number of respondents in each region was calculated according to national population statistical data, taking into consideration the population density in the regions. Furthermore, the sample was proportional to the number of people living in big cities, cities, and villages. When calculating the sample size, sampling design was taken into account.

In each location, where the study was performed, the multiple starting points were randomly selected. The number of starting points depended on the size of the location. In total, 105 starting points were selected. The interviewer would visit every third house. After visiting every third house in the street, they would turn right, and again would visit every third house. From each house, the interviewer would choose one stairwell, usually the first (or where one would manage to enter) and visit every third apartment (household). On average, 10 smokers were interviewed in one cluster.

Within each randomly selected household, the interviewer informed the first adult individual about the purpose of the study, and all current adult smokers were identified. If no adult smokers resided, the interviewer continued to the next household. Otherwise, an adult smoker who had the most recent birthday was selected to complete the questionnaire. Only one individual per household was sampled. In total, 64% of selected households participated in the survey.

In total, 1050 smokers participated in the survey. The characteristics of the participants are presented in Table 1.

According to the Order V-28/2016 of the Minister of Health on the procedure for obtaining the informed consent, written consents are only required for the surveys falling under the biomedical research category. Additionally, according to the Bioethics Center at Lithuanian University of Health Sciences, if the questionnaire is fully anonymous, respondents are not identified, and if objective health information is not collected, written consent is not required.

### 2.2. Questionnaire

The Questionnaire consisting of 17 questions was designed by the researchers of Health Research Institute and was compiled on the basis of previously published smoker survey [23]. Socio-demographic data collected included age, gender, highest education received, area of residence, and current average monthly income. Respondents were classified into two age groups using the median age as the cut-off point (median age = 44). Respondents were divided into higher and lower income groups according to their average monthly income being below (or equal) or above national minimum net wage in Lithuania (€395 as of 2019).

The use of tobacco products was determined using question: “Please list all tobacco or related products that you used during the past 12 month”, with answer options: (1) cigarettes, (2) e-cigarettes, (3) heat-not-burn tobacco products, (4) roll-your-own cigarettes, (5) hookah, (6) cigars and cigarillo, (7) chewing or snuff tobacco, (8) other (specify).

The smoking intensity was measured with the question: “How many cigarettes do you usually smoke per day?” Smokers were divided into two groups by the number of smoked cigarettes: light smokers (1–19 cig/day) and heavy smokers (≥20 cig/day). Smoking intensity classification was based on previously published literature [24,25,26].

Purchasing illicit tobacco was inquired with the question: ”Have you ever bought illicit tobacco products?” Possible answer choices were the following: (1) no; (2) yes, but only sometimes; (3) yes, regularly. Respondents were also asked: “Do you know where you can buy illicit tobacco products?” Answer options were the following: (1) I do not know; (2) I do not know, but it is easy to find out; (3) yes, I know specific places or people I could buy from. The opinion about illicit trade was determined by the question: “What is your overall opinion on the purchase of illicit tobacco products?”, with four response options: (1) positive, I do not see anything wrong with that; (2) negative, but I understand why people buy and use illicit tobacco products; (3) very negative, I would never buy illicit tobacco products myself; (4) I have no opinion.

### 2.3. Identification of Illicit Packs

Identification of illicit cigarette packs was based on the information provided by smokers who either agreed to show the latest pack purchased to the interviewer or provided information about it if they did not have the pack or did not agree to show it.

In both cases, interviewers filled in observational questions to record pack characteristics: (1) the package type (standard 20 cigarette package, roll your own tobacco package, e-device package); (2) health warnings (in Lithuanian, in a foreign language, no health warnings); (3) tax stamps (in Lithuanian, in a foreign language, too damaged to identify language but present, no tax stamp/duty-free pack); (4) price of the pack; and (5) type of outlet/source from where the pack was purchased. A pack could be bought from: (1) national legal shop, kiosk, or catering company; (2) specialized tobacco shop; (3) over the internet; (4) from legal shops in other countries; (5) from duty-free shops; (6) fom individuals selling cigarettes independently at local markets; (7) from individuals when meeting at the time and place agreed; (8) delivered to the house; (9) offered by peers, friends. All abovementioned characteristics later helped to indicate if the pack was illicit.

Following the existing practice from previous study [19], pack was classified as illicit if it had at least one of the four characteristics: (1) pack had no appropriate health warnings—health warning was provided in a foreign language or was not existent (unless the pack had been bought in other country or in a duty-free shop); (2) pack had no appropriate tax stamp—another language than Lithuanian on a tax stamp (unless the pack had been bought in other country or in a duty-free shop), but if the stamp was torn off or damaged it was not considered as illicit; (3) pack with a price lower than the 70% of the lowest price of cigarettes (€3.15) as listed in the WHO country profile 2019 [27] (if not bought over the internet, in other countries or in duty-free shops or offered); (4) pack was bought from an illicit source. In this study, packs purchased over the internet, from individuals at local markets, place agreed or delivered to the house were considered as illicit packs. Regarding the price for a pack of cigarettes, items costing €2.21 and less were considered illicit.

### 2.4. Data and Statistical Analysis

Statistical data analysis was performed using the statistical package IBM SPSS Statistics for Windows, Version 20.0 (2011 version, IBM Corp, Armonk, NY, US) and Microsoft Excel 2019 (Microsoft, Redmond, WA, US). Statistical significance level (*p* values) of 0.05 was chosen to test the hypotheses. The qualitative variables were presented as percentages. Differences in qualitative variables were compared using Chi-squared (*χ2*) and Z test with Bonferroni correction. Student’s t-test was used to compare the mean values of a number of cigarettes smoked per day.

Multivariable logistic regression analysis was used to assess the associations of self-reported buying of illicit cigarettes with socio-demographic variables.

The following criteria were taken into account when approximating the number of illicit cigarettes smoked per year: (1) respondent was smoking cigarettes; (2) respondent reported the number of cigarettes smoked per day; (3) respondent indicated whether he/she has ever bought illicit tobacco products; (4) respondent stated the proportion of illicit cigarettes smoked in the last 12 months. Number of cigarettes smoked per year was calculated by multiplying number of cigarettes smoked per day by 365. Then, number of illicit cigarettes smoked per year was calculated by multiplying number of cigarettes smoked per day by 365 and by the self-reported proportion of illicit cigarettes smoked in the last 12 months. In total, 731 cigarette smokers met abovementioned criteria.

## 3. Results

Table 1 shows the characteristics of the study population. A total of 1050 smokers aged 18–82 years participated in the survey. The mean of respondents’ age was 41.98 (SD 15.35). The most of respondents were younger than 45 years, had higher than secondary education, were living in big cities and had monthly income higher than the minimum net wage. Unfortunately, not all the study respondents were willing to provide personal information about the age, education, or monthly income.

Of all current smokers, 73.7% reported smoking only cigarettes, 5% only e-cigarettes, and 10.7% only heat-not-burn tobacco products. Although cigarette use was more prevalent among men (75.8%), e-cigarettes and/or heat-not-burn tobacco products were more common among women (23.7%) (Table 1).

Table 2 shows the distribution of illicit tobacco purchase by socio-demographic characteristics. Compared to men, a significantly larger proportion of women responded that they had never bought illicit tobacco products. Besides, 13.4% of ≥45 years old individuals regularly buy illicit tobacco products, which is significantly higher proportion compared to 18–44 years old smokers. Respondents with secondary or lower education were more likely to buy illicit tobacco products on a regular basis. A larger proportion of people living in towns or villages regularly bought illicit tobacco products compared to respondents living in big cities in Lithuania. The purchase of illicit tobacco products was more common among lower-income respondents. Five percent of light smokers reported using illicit tobacco products regularly. However, among heavy smokers, the proportion was 3 times higher—15.3%.

The multivariable logistic regression analysis revealed that the odds of self-reported purchase of illicit tobacco products sometimes or regularly were more than two times higher for smokers with secondary or lower education compared to higher than secondary education (Table 3). Likewise, heavy smokers were two times more likely to buy illicit tobacco products sometimes or regularly compared to light smokers.

The odds of regular purchase of illicit tobacco products were 3.04 times higher for 45 and older smokers compared to younger smokers. Having secondary or lower education increased the likelihood of buying illicit tobacco products regularly four times compared to smokers with higher than secondary education. Heavy smokers were 3.61 times more likely to regularly buy illicit tobacco products than light smokers.

Illicit share of the total consumption of cigarettes is based on the self-reported intensity of smoking. The estimated proportion of illicit cigarettes was 10.7% (Table 4). Smoking illicit cigarettes was more common among men. In addition, older age, smoking intensity, lower education, and income level, as well as living in a smaller living place have been associated with consumption of a larger proportion of illicit cigarettes (*p* < 0.001).

Significantly more illicit cigarettes users than non-users (84.4% compared to 28.2%) knew where to acquire illicit tobacco products (Table 5). Attitudes towards purchasing illicit tobacco products differed between those who do or do not purchase it themselves. Significantly fewer smokers of legal tobacco products found nothing wrong with purchasing illicit products (11.3% compared to 53.7%), whereas 3% of smokers who have purchased illicit cigarettes had very negative opinion on the illicit tobacco products compared to more than one third of legal tobacco smokers.

Among the 1050 respondents, the percentage of smokers who showed their packs was relatively low (35.2%, *n* = 370). More than third of respondents (39.5%, *n* = 415) agreed to provide self-reported information on the latest pack bought. Overall, 785 smokers have showed or described the latest pack; however, some packs were excluded from the analysis because either the respondent smoked only other types of tobacco products than cigarettes or did not provide information about all four pack characteristics. Thus, pack observation analysis was performed for 473 packs. As current cigarette smokers reported, 90.9% of those packs were bought from legal tobacco shop in Lithuania, 0.7% from a legal shop in another country, 0.4% from duty-free shops, 7.6% from illicit sources, and 0.4% were offered/gifted by others.

Seven percent of smokers reported an extremely low price of the latest pack (Table 6). The average price of a legal cigarette pack was €3.77 ± 0.39 (with lowest price €2.90 and highest €6.50). The average price of a self-reported illicit cigarette pack was €2.12 ± 0.92 (with lowest price €1.10 and highest €5.10). Furthermore, 9.1% smokers showed or reported packages with and inappropriate health warnings, 7.8%—inappropriate tax stamp. Overall, the prevalence of illicit packs was 9.7%.

Most respondents who reported regularly buying illicit tobacco products and only few of those who bought them sometimes have shown or described illicit cigarette packs (Table 7).

## 4. Discussion

Lithuania has long been listed among countries with the highest prevalence of illicit cigarettes in the EU. To our knowledge, this paper is the first to provide industry-independent nationally representative estimates on the illicit cigarette market in Lithuania. The results of our survey indicate that illicit share of the total consumption of cigarettes per year was 10.7% with 9.7% of smokers showing or reporting illicit cigarette packs compared to 17% reported by industry-funded studies. Smokers with lower education, 45 years old or older, and heavy smokers were more likely to regularly purchase illicit cigarettes. Additionally, older age, being male, smoking intensity, lower education, and income level, as well as living in a smaller living place have been associated with the consumption of a larger proportion of illicit cigarettes. The average price of illicit packs of cigarettes was almost two times lower than licit.

The illicit market of tobacco products in Lithuania has always been a heated argument between public health society and TI allies. Nevertheless, industry-independent studies on illicit tobacco in Lithuania are scarce. Most often estimates of the illicit trade are provided by TI-related and/or funded organizations (i.e., KPMG, Euromonitor, Transcrime, Lithuanian Free Market Institute).

Evaluation of industry-funded studies conclude that there are some major issues regarding the quality of the estimates, including lack of methodological transparency; problems with data collection, analytical methods and result interpretation; and that they are not peer-reviewed [10,28]. Some studies show that industry-driven data exaggerate the extent of illicit trade [3,10,28,29,30,31,32,33,34,35]. It appears that our findings support this observation.

As previously mentioned, the latest (2018) KPMG report shows that Lithuania had one of the highest shares (17%) of the counterfeit and contraband cigarettes in the EU [18]. According to our estimates, share of illicit cigarettes is 1.5 times lower than industry estimates. Thus, a direct comparison of these estimates is very complex.

PMI IMPACT (an initiative of Philip Morris International Inc) funded survey performed by Sprinter and commissioned by the Lithuanian Free Market Institute showed that 17% of respondents self-reported purchase of cigarettes from illicit sources in the past year in 2018 [36]. Thus, we found that 11.6% of smokers reported purchasing illicit cigarettes only sometimes in their lifetime, and 6.8% reported doing so regularly. Again, it is hard to directly compare estimates, as methodological aspects of industry-funded studies mostly remain unknown.

According to another survey performed by Sprinter and commissioned by the State Tax Inspectorate, Lithuanians assume that the share of the illicit tobacco trade accounted for 16.3% in 2017 [37]. Our estimates are lower. Arguably, surveys that analyse attitudes and perceptions of citizens and do not ask about their specific smoking habits cannot be compared and should not be considered the same as methods directly evaluating the illicit share of the tobacco.

At the same time, our estimates of the share of illicit packages (9.7%) are comparable with the overall proportion (6.5%) of illicit cigarettes and hand-rolled tobacco in 18 European countries, as estimated in the PPACTE survey in 2010 [19]. Some previous research stated that 9.8% of cigarette consumption in high-income countries is illicit [7].

Findings from our study confirmed that, as in the previous studies, older smokers [29,33,38], smokers who smoke more intensively [3,34,38] and have lower education [3,19,33,34,38,39] are more likely to use the illicit cigarettes.

Our analysis barely detected any cases of tax-avoidance. Only a few (1%) smokers in Lithuania reported buying tax-avoided cigarettes from duty-free shops or legal shops abroad. This is consistent with overall patterns in the EU, in an average EU Member State only a small portion of cigarette sales can be explained by cross-border purchasing [40]; besides, in most EU countries only a small portion of all smokers reported frequent cross-border cigarette purchasing [41,42]. Evidence suggests that smokers living in regions bordering countries where cigarettes were at least €1 per pack lower than in their home country had significantly higher odds of purchasing cheaper out-of-country cigarettes [43]. However, due to the tax harmonization policy in the EU, the weighted average price of cigarettes in Lithuania, Latvia, and Poland are almost the same, €3.18, €3.20, and €3.26 per pack respectively [44]. At the same time, in Russia and Belarus, cigarette prices are almost 3 times lower. Nonetheless, cross-border shopping even for the smokers living close to the physical borders with the later countries might be inconvenient due to required visas.

This study provides industry-independent estimates on the illicit cigarette consumption in Lithuania and is the first to counterbalance existing TI-related evidence. The results of this study may assist policy makers to revise tobacco taxation and approaches for controlling illicit tobacco trade. Another strength of the analysis is that we followed the recent practice [3,13,19,29,31,33,34,39,45,46] to validate self-reported information with observational pack data by asking to show the latest purchased cigarette pack and inspecting it.

Unfortunately, the percentage (35.2%) of smokers who showed their packs was low. Compared to other studies, the percentage was higher than in Mexico (29%) [3] but smaller than in Turkey (76%) [39], Georgia (71%) [47], and average from PPACTE study of 18 European countries (74%) [19]. It is difficult to explain the reasons for such a low proportion of shown packages. Smokers’ unwillingness to show pack may be related to the desire to complete the survey as soon as possible and simply choosing a more convenient option—to describe the package rather than to go and look for it. On the other hand, smokers who reported never purchasing illicit cigarettes were more likely to show the illicit pack. This might indicate that smokers who had illicit packs did not feel comfortable to admit it. In this case, we could have underestimated the proportion of illicit packs.

There is a long and well-described tradition of the TI to use the threat of the illicit trade to compromise such evidence-based policies as tobacco tax and price regulations [5,48,49,50,51,52], plain packaging [32,53], pictorial warnings [49], point-of-sale display bans [49], marketing regulations [54], flavour bans [49,55], pack size restrictions [56]. TI in particular often claims that taxes on tobacco products drive the illicit trade [5,57,58,59,60,61], although there is a vast amount of evidence showing a limited relationship between higher tobacco prices and size of the illicit market [14,62,63,64,65,66]. In fact, illicit trade can be minimized even in the presence of increased taxes if effective monitoring and enforcement is in place [67,68,69].

Furthermore, Shafferer et al. [69] estimated that tax-induced price hikes of 10% would increase illicit cigarette use the most (by 11%) in Czech Republic, Latvia, Lithuania, Poland, and Slovakia out of the 36 European countries. At the same time, the consumption of licit cigarettes in these countries would be reduced by 18.4%. The average tax loss due to price increases in all the observed 36 European countries was estimated to account for about $377.46 million USD, whereas average total tax increases could be as high as $14.69 billion USD. This shows that tax losses would be marginal in comparison with the total gains in taxes.

Lowering overall demand is an evidence-based strategy of reducing illicit trade and taxation is one of the most effective measures to reduce demand [41]. According to the European Commission, the weighted average price of cigarettes in Lithuania is still one of the lowest in the EU (€3.18 per pack; cheaper cigarettes can be found only in Bulgaria—€2.57) [44]. What is more, among 36 European countries, Lithuania scores 29th in the Tobacco Control Scale 2019. The Tobacco Control Scale report states that in Lithuania “fear of illicit supply of the cigarettes from neighbouring countries and tobacco industry pressure contribute to tax levels remaining low” [70]. Given the fact that tobacco taxation is the most effective measure to reduce smoking and the growth of the illicit market is the most common contra-argument, lack of reliable data on illicit tobacco allows speculative positions and threatens the country’s efforts to reduce smoking.

The study was subject to limitations. First, the selected study design might have caused selection bias. However, the distribution of the selected smokers by sociodemographic characteristics did not differ from that in the Health Behaviour among Lithuanian Adult Population study where all participants were randomly selected from the National population registry [71]. We did not account for clustering in the data analysis. Therefore, some *p*-values might be too low, and the risk of a false-positive error increased. However, a high number of clusters and a small number of respondents in the clusters, as well as a long distance between households where smokers were interviewed might have reduced both the interaction between respondents and the cluster effect.

Second, our survey provides most estimates based on self-reporting, and consequently likely to be under-estimated. However, direct observation of smokers’ packs or asking concrete questions about the pack is likely to elicit responses that are both honest and less subject to respondent error [32]. There may be errors when respondents do not accurately remember the price paid or other details. Smokers may not answer truthfully about the number of cigarettes smoked. This is especially valid if smokers are questioned in the household where other family members might be present. Following the example of other studies [29,33,34], the latter situations might be avoided if smokers are questioned in the street.

Third, we assume that the latest pack purchased reflects characteristics of the usual pack. This assumption is standard and relies on smokers’ loyalty to particular brands. Nevertheless, in the future surveys it is possible to ask to show all packages that smokers have at home. Besides, the previous study in Poland, showed that if asked to show all packs in their possession, smokers show both, taxed and non-taxed packs [13].

Fourth, we cannot differentiate between packs intended and not intended for the domestic market. Fifth, our data on the last purchased pack did not include a brand name, which could have allowed us to better validate self-reported information and identify “cheap whites” in the Lithuanian market. Sixth, the relatively small size of the database and sometimes lack of complete survey response could have had an impact on the results. More extensive data could have enabled a more rigorous statistical analysis to have been undertaken from which more reliable conclusions could have been generated. The current study deserves further confirmatory investigations with a major focus on data collection.

Survey of smokers’ packs could be improved by increasing the sample size, assuring better training for interviewers, and having the possibility to take pictures of the pack that actually would strengthen cross-validation of data.

The International Agency for Research on Cancer identified three methods that are most used to measure illicit trade: (1) comparison of tax paid sales and individually reported consumption measures, also called gap analysis; (2) survey of tobacco users’ purchase behaviours; and (3) observational data (empty packs) collection [68]. This paper describes only one of the methods used to determine the illicit trade of cigarettes, though some authors [3,13] suggest that the most optimal practice for researchers could be cross validation of estimates using both, survey and the littered pack survey.

Due to the low number of packs observed, there was no comprehensive analysis of regional differences in illicit cigarettes consumption. Previous research shows that smokers in border regions are more likely to engage in cross-border purchases from neighbouring countries with lower tobacco prices [40,43,72]. Similarly, more illicit cigarettes are found in the living places close to borders [38,45]. Current evidence does not allow us to evaluate if illicit packs were more common in the areas closer to borders.

Therefore, further independent, and comprehensive data on illicit tobacco could address the data gaps by applying mixed methods, providing data across population groups and geographies, as well as estimate taxes lost through illicit trade. Besides, the survey showed that the proportion of smokers who smoke only e-cigarettes or heat-not-burn cigarettes, as well as the proportion of dual smokers, is relatively high. Further research on the consumption of other tobacco products is needed.

## 5. Conclusions

This study provides TI-independent and methodologically transparent estimates of illicit trade in Lithuania, showing the lower share of illicit trade than reported by industry-funded studies. Although the illicit trade of tobacco products is a serious policy challenge in Lithuania, the threat of an increase in illicit trade should not delay reinforcement of the implementation of the WHO recommended tobacco control policies (MPOWER), and tobacco taxation policies in particular. Further research employing mixed methods would be needed to better assess the magnitude of the illicit tobacco trade.

## Figures and Tables

**Table 1:** Characteristics of the study population.

**Characteristic**	**Men (%)**	**Women (%)**	**Total (%)**	***p* between Men and Women**
**Total *n* = 1050**	73.4	26.6	100	
**Age groups *n* = 692**				0.199
18–44	55.4	61.1	56.8	
≥45	44.6	38.9	43.2	
**Education *n* = 643**				0.011
Secondary or lower	41.9	30.7	39.0	
Higher than secondary	58.1	69.3	61.0	
**Living place *n* = 1050**				0.202
Big city (from 91,000 residents)	47.2	41.6	45.7	
City (2000–91,000 residents)	20.9	21.2	21.0	
Town/village (up to 2000 residents)	31.9	37.2	33.3	
**Monthly income *n* = 479**				0.009
≤€395	25.1	37.6	28.2	
>€395	74.9	62.4	71.8	
**Tobacco products used *n* = 1050**				
Cigarettes only	75.8	68.0	73.7	0.011
Cigarettes and other tobacco products	10.0	7.2	9.2	0.102
Only e-cigarettes	4.2	7.2	5.0	0.051
Only heat-not-burn tobacco products	8.7	16.2	10.7	0.001
Only e-cigarettes and/or heat-not-burn tobacco products	13.2	23.7	16.0	0.001
E-cigarettes or heat-not-burn tobacco products and cigarettes with other tobacco products	20.7	30.9	23.4	0.001
Only roll-your-own cigarettes, cigars and cigarillos, smokeless tobacco products (chewing tobacco, snuff)	1.1	0.7	1.0	0.626
**Smoking intensity *n* = 965**				<0.001
Light smokers (1–19 cig/day)	74.8	90.2	78.9	
Heavy smokers (≥20 cig/day)	25.2	9.8	21.1	
**The average number of cigarettes smoked per day *n* = 965**	13.7 ± 6.7	10.5 ± 5.1	12.9 ± 6.5	<0.001

**Table 2:** Distribution of illicit tobacco purchase by selected socio-demographic characteristics and smoking intensity.

Characteristic	Have You Ever Bought Illicit Tobacco Products?				*p* from Pearson Chi-Square Test
	Never (%)	Yes, But Only Sometimes (%)	Yes, Regularly (%)	*n*	
**Total**	81.6	11.6	6.8	970	
**Sex**					0.001
Men	78.8 *	13.6 *	7.6	708	
Women	89.3	6.5	4.2	262	
**Age groups**					<0.001
18–44	84.1 *	12.7	3.2 *	377	
≥45	74.7	11.9	13.4	269	
**Education**					<0.001
Secondary or lower	73.7 *	12.7	13.6 *	228	
Higher than secondary	86.0	10.5	3.5	372	
**Living place**					0.046
Big city (from 91,000 residents)	84.2 *	11.3	4.5 *	444	
City (2000–91,000 residents)	83.1	9.7	7.2	207	
Town/village (up to 2000 residents)	77.1	13.5	9.4	319	
**Monthly income**					0.056
≤€395	75.4	9.8	14.8 *	122	
>€395	80.6	12.0	7.4	324	
**Smoking intensity**					
Light smokers (1–19 cig/day)	83.5 *	11.5	5.1 *	707	<0.001
Heavy smokers (≥20 cig/day)	68.9	15.8	15.3	183	

* Statistically significant difference compared to women, having higher than secondary education, living in town or village, having monthly income >€395, and being heavy smoker, respectively (Z test with Bonferroni correction, *p* < 0.05).

**Table 3:** Odds ratios of purchase of illicit tobacco products according to socio-demographic characteristics and smoking intensity.

Variables	Have You Ever Bought Illicit Tobacco Products?					
	Yes, But Only Sometimes/Yes, Regularly			Yes, Regularly		
	OR	95% CI	*p*	OR	95% CI	*p*
Sex						
Men	1			1		
Women	0.57	0.31–1.05	0.073	0.94	0.38–2.33	0.900
Age groups						
18–44	1			1		
≥45	1.43	0.90–2.26	0.133	3.04	1.44–6.431	0.004
Education						
Secondary or lower	1			1		
Higher than secondary	0.46	0.29–0.74	0.001	0.25	0.12–0.51	<0.001
Living place						
Big city (from 91,000 residents)	1			1		
City (2000–91,000 residents)	0.79	0.43–1.45	0.452	0.88	0.34–2.24	0.783
Town/village (up to 2000 residents)	1.29	0.77–2.16	0.330	1.45	0.67–3.12	0.348
Smoking intensity						
Light smokers (1–19 cig/day)	1			1		
Heavy smokers (≥20 cig/day)	2.08	1.24–3.49	0.006	3.61	1.78–7.33	<0.001

OR—odds ratio; CI—confidence interval.

**Table 4:** Number of cigarettes smoked per year and the proportion of illicit cigarettes by socio–demographic characteristics and smoking intensity.

Variables	Number of Cigarettes per Year	Number of Illicit Cigarettes per Year	The Proportion of Illicit Cigarettes out of All Cigarettes (%)	*p* from Z Test
Sex *n* = 731	3,456,185	369,289	10.7	<0.001
Men	2,771,445	317,714	11.5	
Women	684,740	51,575	7.5	
Age groups *n* = 486				<0.001
18–44	1,081,130	73,311	6.8	
≥45	1,243,190	184,599	14.8	
Education *n* = 444				<0.001
Secondary or lower	837,310	174,744	20.9	
Higher than secondary	1,284,435	83,476	6.5	
Living place *n* = 731				<0.001
Big city (from 91,000 residents)	1,388,825	114,920	8.3	
City (2000–91,000 residents)	815,410	87,600	10.7	
Town/village (up to 2000 residents)	1,251,950	166,769	13.3	
Monthly income *n* = 318				
≤€395	288,715	81,778	28.3	<0.001
>€395	1,231,875	152,552	12.4	
Smoking intensity *n* = 731				<0.001
Light smokers (1–19 cig/day)	2,160,070	154,304	7.1	
Heavy smokers (≥20 cig/day)	1,296,115	214,985	16.6	

**Table 5:** Distribution of respondents by knowing where to buy illicit tobacco products and opinion on the purchase according to purchase history of illicit tobacco products.

Survey Questions about Knowledge and Attitudes on Illicit Tobacco Products	Have You Ever Bought Illicit Tobacco Products?	
	No (%) *n* = 717	Yes (Yes, But Only Sometimes/Yes, Regularly) (%) *n* = 164
Do you know where you can buy illicit tobacco products?		
I do not know;	71.8	15.6
I do not know, but it is easy to find out; Yes, I know specific places or people I could buy from.	28.2 *	84.4
What is your overall opinion on the purchase of illicit tobacco products?		
Positive, I do not see anything wrong with that;	11.3 *	53.7
Negative, but I understand why people buy and use illicit tobacco products;	39.9	36.6
Very negative, I would never buy illicit tobacco products myself;	34.7 *	3.0
I have no opinion.	14.1 *	6.7

* Statistically significant difference compared to respondents who bought illicit tobacco products (Z test with Bonferroni correction *p* < 0.05), Pearson Chi-Square *p* < 0.001.

**Table 6:** Proportion of smokers who showed or reported illicit cigarette packs according to the selected criteria.

Variables	*N* of Smokers	1. Self-Reported Purchase from Illicit Sources (%)	2. Inappropriate Health Warning (%)	3. Inappropriate Tax Stamp (%)	4. Extremely Low Price (%)	5. Illicit Pack (at Least One out of the Four Criteria) (%)
**Pack shown**	279	6.5	7.2	6.1	5.4	7.9
**Pack described**	194	9.3	11.9	10.3	9.3	12.4
**Total**	473	7.6	9.1	7.8	7.0	9.7

**Table 7:** Proportion of smokers who showed or described illicit cigarette packs according to self-reported frequency of purchasing illicit tobacco products.

Variables	Have You Ever Bought Illicit Tobacco Products?	
	Yes, But Only Sometimes (%)	Yes, Regularly (%)
**Pack shown**	2.9	88.2
**Pack described**	0	100
**Total**	1.4	94.4
